# Topical Administration of a Marine Oil Rich in Pro-Resolving Lipid Mediators Accelerates Wound Healing in Diabetic *db/db* Mice through Angiogenesis and Macrophage Polarization

**DOI:** 10.3390/ijms23179918

**Published:** 2022-08-31

**Authors:** Imelda Ontoria-Oviedo, Elena Amaro-Prellezo, Delia Castellano, Elena Venegas-Venegas, Fernando González-Santos, Amparo Ruiz-Saurí, Beatriz Pelacho, Felipe Prósper, María Dolores Pérez del Caz, Pilar Sepúlveda

**Affiliations:** 1 Regenerative Medicine and Heart Transplantation Unit, Instituto de Investigación Sanitaria La Fe, 46026 Valencia, Spain; 2 Solutex GC, S.L., Parque Empresarial Utebo, 50180 Utebo, Spain; 3 Departamento de Patología, Facultad de Medicina, Instituto de Investigación Sanitaria INCLIVA, Universitat de Valencia, 46026 Valencia, Spain; 4 Regenerative Medicine Department, Center for Applied Medical Research (CIMA), Instituto de Investigación Sanitaria de Navarra (IdiSNA), University of Navarra, 31008 Pamplona, Spain; 5 Departamento de Hematología y Terapia Celular, Clínica Universidad de Navarra, 31008 Pamplona, Spain; 6 CIBERCV, Carlos III Institute of Health, 28029 Madrid, Spain

**Keywords:** pro-resolving lipid mediators, wound healing, omega-3, macrophage polarization, angiogenesis, SPMs, diabetic ulcer

## Abstract

Impaired wound healing in patients with type 2 diabetes (DM2) is characterized by chronic inflammation, which delays wound closure. Specialized pro-resolving lipid mediators (SPMs) are bioactive molecules produced from essential polyunsaturated fatty acids (PUFAs), principally omega-3 docosahexaenoic acid (DHA) and eicosapentaenoic acid (EPA). SPMs are potent regulators of inflammation and have been used to suppress chronic inflammation in peripheral artery disease, non-alcoholic fatty liver disease, and central nervous system syndromes. LIPINOVA^®^ is a commercially available safe-grade nutritional supplement made from a fractionated marine lipid concentrate derived from anchovy and sardine oil that is rich in SPMs and EPA, as well as DHA precursors. Here, we assessed the effect of LIPINOVA^®^ in wound dressing applications. LIPINOVA^®^ showed biocompatibility with keratinocytes and fibroblasts, reduced the abundance of pro-inflammatory macrophages (Mφ1), and promoted in vitro wound closure. Daily application of the marine oil to open wounds made by punch biopsy in *db/db* mice promoted wound closure by accelerating the resolution of inflammation, inducing neoangiogenesis and Mφ1/Mφ2 macrophage polarization. In conclusion, LIPINOVA^®^ displays pro-resolutive properties and could be exploited as a therapeutic agent for the treatment of diabetic ulcers.

## 1. Introduction

Wound healing is a complex and dynamic response to mechanical/chemical injury, and the abnormal wound healing (non-healing) processes can lead to chronic wound ulcers [1]. A recent systematic review showed that, among other types of chronic wounds, leg and foot ulcers are the most prevalent in the general population [2], and they are commonly associated with type 2 diabetes (DM2) [3]. DM2, a metabolic disorder caractherized by hyperglycemia due to insuline resistance, is associated with numerous co-morbidities, including skin ulcers. The incidence and prevalence of this disease has reached very high values, becoming a major global health epidemic [4,5]. Wound ulcers caused by DM2 affect aproximately 15% of diabetic patients and are a leading cause of amputations [6]. DM2 ulcers have an immense social and economic impact on people and health systems due to their high incidence, especially among the elderly with coexisting risk factors, such as hypertension or smoking. Mechanistically, wound healing involves the coordinated response of several cell types of the dermis and epidermis, which is achieved through four different temporally- and spatially-distinct but overlapping phases: hemostasis, inflammation, proliferation, and remodeling [7]. In the setting of chronic wounds, progression between the different phases is compromised, and the resolution phase is not reached, leading to a prolonged inflammatory state [8].

Different strategies have been tested for the treatment of diabetic ulcers, ranging from simple and low-cost approaches that include cleansing, debridement, and maintenance of moisture balance with wound dressings, to more sophisticated approaches, such as the use of mesenchymal stem cells [9], or the application of complete skin grafts [7]. Beyond cellular-based mechanisms, the potential utility of bioactive omega-3 (ω-3) polyunsaturated fatty acids (PUFAs), including eicosapentaenoic (EPA) and docosahexaenoic (DHA) acids for wound healing, is under intense investigation [10,11,12].

ω-3 PUFAs are essential fatty acids that must be obtained from the diet [13], with the main sources being plants and cold water fish oil [14]. Interest in ω-3 PUFAs has risen in recent years because of their beneficial effects in the resolution of inflammation in the context of different pathological conditions [15]. For example, administration of ω-3 PUFAs reduces circulating triglyceride levels and body weight in people with obesity [16,17]. They also have demonstrated anti-fibrogenic activity in patients with liver disease [18], and can modulate pro-inflammatory responses in patients with vascular disease [19] or chronic degenerative diseases such as Alzheimer’s disease and multiple sclerosis [20]. By contrast, the effects of ω-3 PUFAs in wound healing are less well studied and are controversial [21,22]. For example, the addition of DHA and EPA to cultures of human microvascular endothelial cells was shown to suppress proliferation and migration in an in vitro scratch assay [23]. In another study, ω-3 PUFAs were proven to affect the gene expression of pro-inflammatory cytokines and modulate wound healing when taken orally; however, no differences were found in healing in a cohort of subjects receiving a ω-3 fatty acid EPA/DHA (fish oil) supplement for 4 weeks when compared with a placebo cohort [24]. Nonetheless, other authors have observed benefits of oral supplementation of ω-3 PUFAs in wound-mediated infections [25,26]. Moreover, oral supplementation of EPA and DHA was found to be beneficial in the non-surgical management of periodontitis [27], and a body of evidence indicates a positive role of ω-3 PUFAs in preventing the progression of periodontal disease [28].

EPA and DHA are known to exert immunoregulatory effects that can promote the resolution of inflammation [13,29]. Together with arachidonic acid (AA), they serve as substrates for the biosynthesis of pro-resolving lipid mediators (SPMs), which are organized into several structurally distinct families termed lipoxins, resolvins, protectins, and maresins. SPMs are produced by different subtypes of macrophages, neutrophils, and exudate cells, and they promote wound healing and tissue regeneration [30,31]. Intermediate metabolites between these substrates and the resolvins, protectins and maresins families are monohydroxylated 18-hydroxyeicosapentaenoic acid (18-HEPE) and the hydroxy docosahexaenoic acids 17-HDHA and 14-HDHA [32], which have been identified in human fluids including serum, plasma, urine, and breast milk [33]. Interestingly, oral supplementation of fish oil increases the levels of these SPMs in patients with peripheral arterial disease (PAD) [34]. Beneficial effects of single molecules of these families or their substrates, for example, LXA4 (derived from AA) or resolvin D1 and D2 (derived from DHA), have been reported in animal models with different pathologies, including wound healing [25,35,36,37,38]. However, SPMs display non-redundant functions on different target cells, and thus it is difficult to envision that a single molecule could be sufficient to induce a complex pro-resolutive healing process in a clinical setting.

Recently, SPM extracts have been standardized in marine oil fractions from anchovy and sardine skin, and commercially formulated LIPINOVA^®^ is available from Solutex GC (https://www.solutex.es/; Parque Empresarial Omega, Edificio Gamma, Avenida de la Transición Española 24, 3ª 28108 Alcobendas, Spain). LIPINOVA^®^ is enriched with ω-3 fatty acids and contains several SPMs, including 18-HEPE, 17-HDHA, and 14-HDHA. When administered orally to patients with PAD, LIPINOVA^®^ influenced circulating levels of SPMs and modulated the immune system to pro-resolutive phenotypes, promoting improvements in inflammation [39,40]. The same nutritional supplement was also used successfully to reduce chronic pain and improve quality of life in patients with chronic pain, depression, and anxiety (ClinicalTrials.gov, (accessed on 14 July 2022)); Influence of an Omega-3 SPM Supplement on Quality of Life (NCT02683850) [41].

In the present study, we evaluated the therapeutic potential of LIPINOVA^®^ as a topical application for diabetic ulcers, which are often cavernous open wounds that would benefit from wound dressing formulations. We found that LIPINOVA^®^ promotes fibroblast and keratinocyte migration; is internalized by fibroblasts, macrophages, and endothelial cells; and reduces the levels of macrophages with an inflammatory (Mφ1) phenotype. LIPINOVA^®^ also accelerated open wound closure, increased blood vessel density, and promoted anti-inflammatory Mφ2 polarization when applied daily to open wounds in *db/db* mice.

## 2. Results

### 2.1. Study Design and Biocompatibility of LIPINOVA^®^ In Vitro

**Figure 1:**
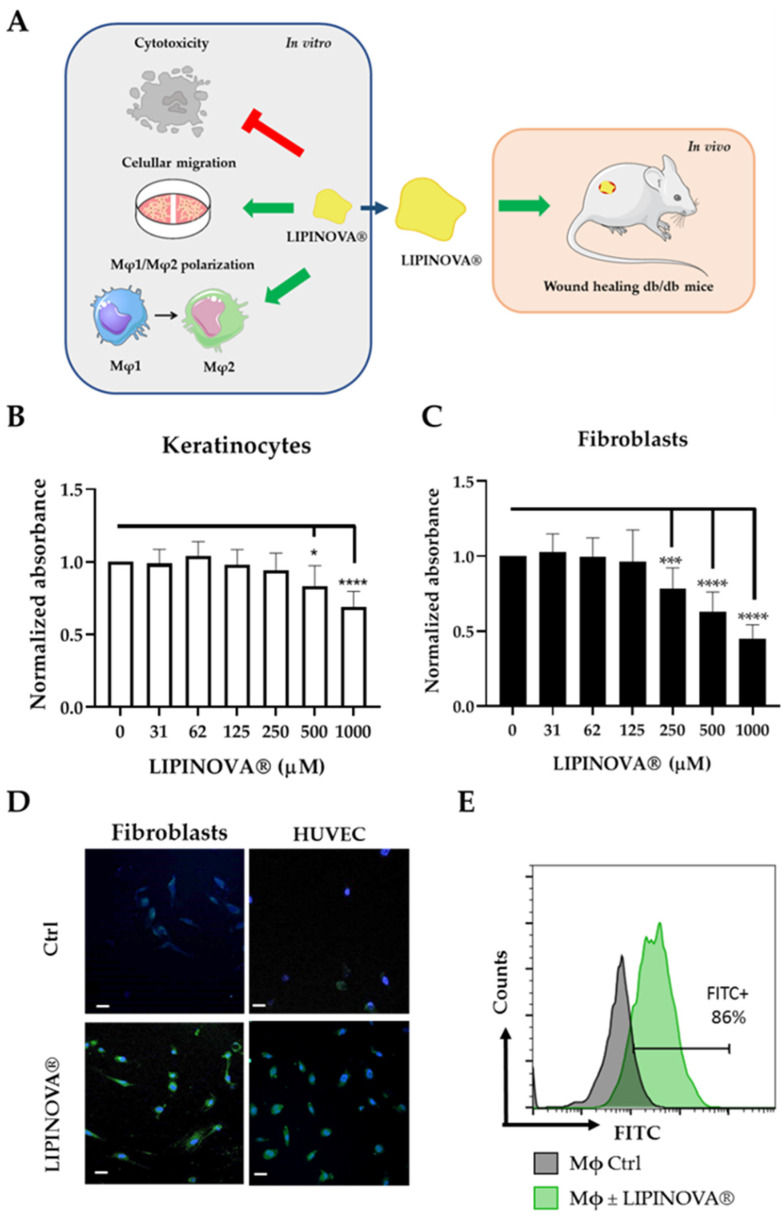
Keratinocyte and fibroblast viability as a function of LIPINOVA^®^ concentration. (**A**) Schematic design of the study. LIPINOVA^®^ was assessed in vitro for cytotoxicity; uptake by dermal fibroblasts, endothelial cells, and macrophages; scratch wound healing; and macrophage polarization. Thereafter, the therapeutic potential of LIPINOVA^®^ was measured in an in vivo wound healing assay in *db/db* mice. Viability of (**B**) keratinocytes and (**C**) fibroblasts assessed with the CCK-8 assay after incubation with different concentrations of LIPINOVA^®^. Absorbance was measured at 450 nm, and data are represented as mean ± SD of three independent experiments. Two-way ANOVA was used for statistical analysis. * *p* < 0.05, *** *p* < 0.001, and **** *p* < 0.0001. (**D**) Immunofluorescence analysis of HUVEC after 6 h incubation with 100 µM LIPINOVA^®^-OG or saline (Ctrl). Cells were fixed and stained with DAPI (blue). Images were acquired with a fluorescence microscope with a 20× objective. Scale bar = 50 µm. (**E**) Flow cytometry assay to measure LIPINOVA^®^ internalization in human macrophages.

LIPINOVA^®^ marine oil supplement contains PUFAs, among other fatty acids (Table 1). We determined the concentrations of the SPM precursors and substrates in the supplement batch used in the present study, expressed as μg/15 mL, which is equivalent to a dose of 1.5 g. The extract contained 17-HDHA (179.6 mg/kg), 18-HEPE (278.1 mg/kg), and 14-HDHA (97.7 mg/kg). We then evaluated the resolutive properties of LIPINOVA^®^ in the context of DM2-related ulcers. Specifically, we first analyzed the functional activity of the formulation in vitro using fibroblasts, endothelial cells, and macrophages, and later verified its properties in diabetic (*db*/*db*) mice with open wounds (Figure 1A).

We first evaluated the potential cytotoxicity of LIPINOVA^®^ in primary cultures of keratinocytes and fibroblasts seeded and incubated with different concentrations of LIPINOVA^®^. Cell viability was measured using the CCK-8 assay 48 h after plating. No changes in viability were observed in keratinocytes treated with LIPINOVA^®^ at concentrations equal to or below 250 µM LIPINOVA^®^ (Figure 1B). By contrast, fibroblasts appeared to be more sensitive to LIPINOVA^®^, and concentrations greater than 125 µM significantly reduced viability (Figure 1C). Uptake of LIPINOVA^®^ by dermal fibroblasts and human umbilical vein endothelial cells (HUVEC) was assessed by internalization assays with LIPINOVA^®^ labeled with Oregon green (LIPINOVA^®^-OG). Cells showed an increase in fluorescence when they were incubated with LIPINOVA^®^-OG, as compared with non-treated cells (Figure 1D). The addition of LIPINOVA^®^-OG to human macrophages from peripheral blood also resulted in cellular uptake, as assessed by flow cytometry (Figure 1E).

### 2.2. LIPINOVA^®^ Promotes Migration and Spreading of Fibroblast and Keratinocytes

**Figure 2:**
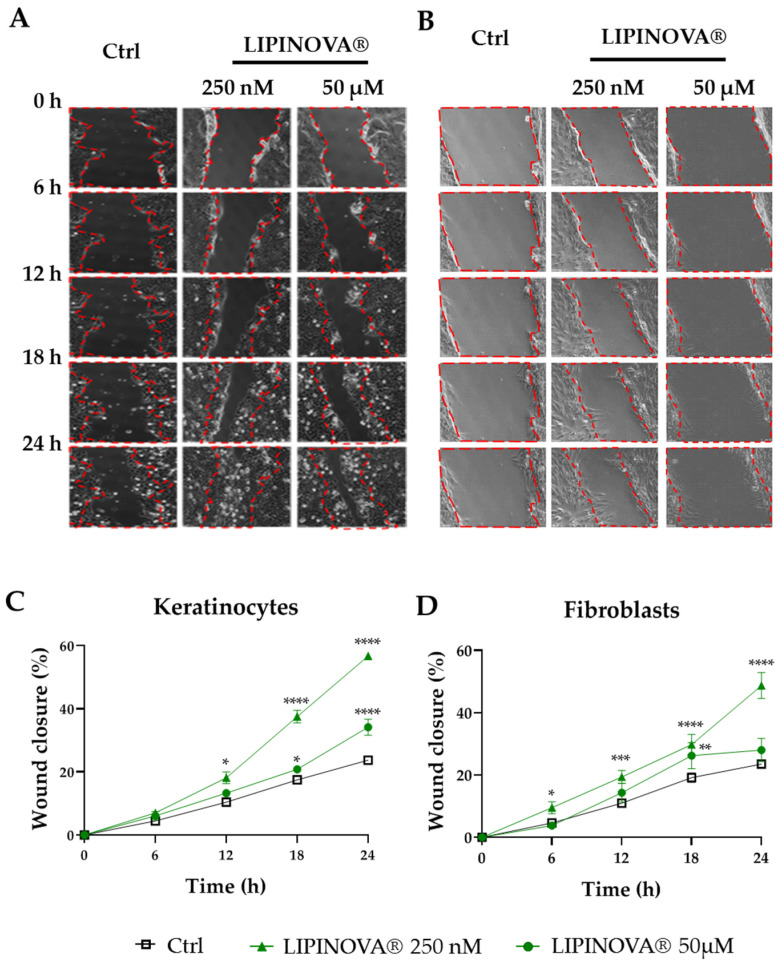
LIPINOVA^®^ treatment accelerates the in vitro migration of keratinocytes and fibroblasts. Representative brightfield images of a scratch assay at different timepoints after scratching a culture plate of keratinocytes (**A**) and fibroblasts (**B**) treated with 250 nM or 50 µM of LIPINOVA^®^, or with saline (Ctrl). Dotted lines define the wound area; scale bar = 100 µm. Quantification of wound closure of keratinocytes (**C**) and fibroblasts (**D**) in the presence of LIPINOVA^®^. Data were normalized to an initial wound area and are represented as mean ± SD percentage. Images were taken at 20× magnification. Experiments were performed in triplicate. Two-way ANOVA was used for statistical analysis. * *p* < 0.05, ** *p* < 0.01, *** *p* < 0.001, and **** *p* < 0.0001.

Wound closure is mediated by re-epithelization and migration of fibroblasts to the wound site. To investigate whether LIPINOVA^®^ can modulate the migration of keratinocytes and fibroblasts, we measured its ability to promote wound closure in an in vitro scratch-wound assay (Figure 2A,B). Based on previous studies evaluating the effects of SPMs and PUFAs on different cell types [29,42], we chose 50 µM LIPINOVA^®^ as the highest concentration. Results showed that LIPINOVA^®^ significantly increased keratinocyte migration at all the concentrations tested (results not shown), with 250 nM LIPINOVA^®^ identified as the most effective concentration when migration was measured 24 h after treatment (Figure 2C). Similar results were observed for fibroblasts (Figure 2D), and the effectiveness of the treatment was evident after only 6 h following the addition of the supplement.

### 2.3. Inflammatory Response to LIPINOVA^®^ In Vitro

**Figure 3:**
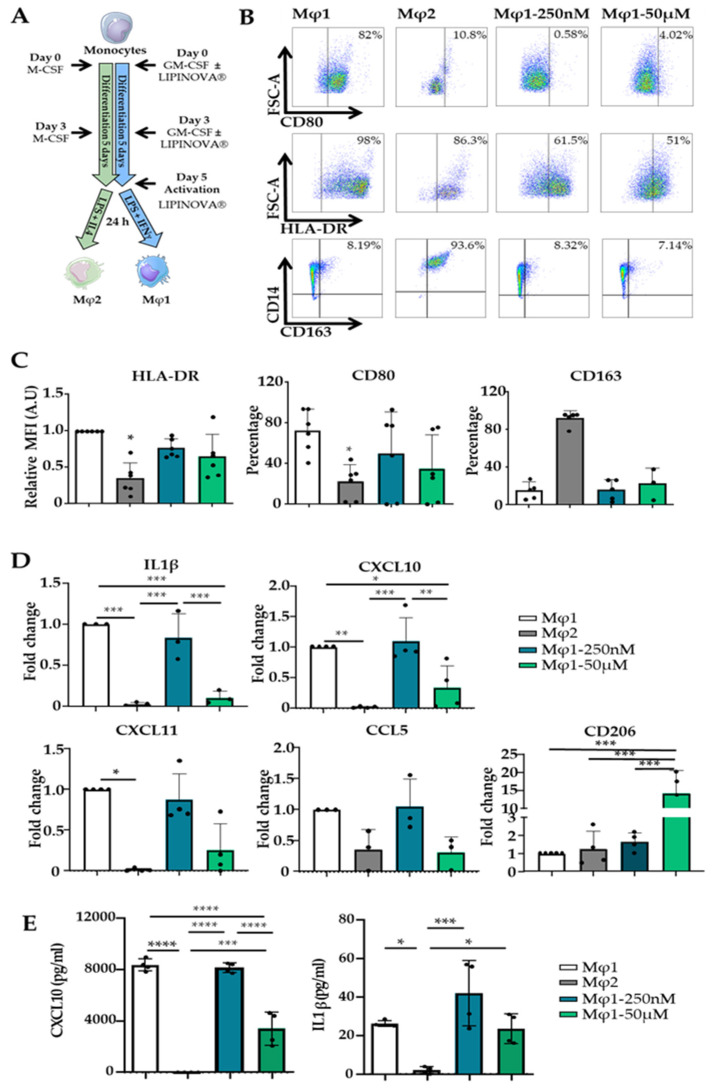
LIPINOVA^®^ modulates the expression of Mφ1 and Mφ2 markers in vitro. (**A**) Differentiation scheme of monocytes using GM-CSF, LPS, and IFNɣ to obtain Mφ1, and M-CSF, LPS, and IL4 to obtain Mφ2. LIPINOVA^®^ was added on days 0, 3, and 5. (**B**) Representative dot plots of HLA-DR+, CD80+, and CD163+ cells assessed by flow cytometry on day 6 after differentiation.(**C**) Graphs representing flow cytometry quantification of Mφ1 (white), Mφ2 (grey), and Mφ1 incubated with LIPINOVA^®^ at 250 nM (cyan) or 50 µM (green). Data are represented as mean ± SD of five independent experiments. (**D**) Gene expression levels of IL1β, CXCL10, CXCL11, and CD206 quantified by qPCR in Mφ1 (white), Mφ2 (grey), and Mφ1 incubated with 250 nM (cyan) or 50 µM (green) LIPINOVA^®^ during differentiation. (**E**) ELISA assay to assess CXCL10 and IL1 β production by Mφ1 (white), Mφ2 (grey), and Mφ1 incubated with 250 nM (cyan) or 50 µM (green) LIPINOVA^®^ at day 6 of differentiation. Data are represented as mean ± SD of four independent experiments. Each dot represent an independent experiment. One-way ANOVA was used for statistical analysis; * *p* < 0.05, ** *p* < 0.01, *** *p* < 0.001, and **** *p* < 0.0001.

Pro-resolving mediators, such as resolvin D1, or their precursors have been shown to promote macrophage polarization towards an Mφ2 phenotype [29]. We next performed an in vitro polarization assay to evaluate the ability of LIPINOVA^®^ to modulate this process. Monocytes were isolated and differentiated to Mφ1 and Mφ2 (see methods). During the course of differentiation to Mφ1, some cultures were treated with 250 nM or 50 µM LIPINOVA^®^, which was freshly added every 3 days, and surface markers were compared with non-treated Mφ1 and Mφ2 differentiated populations (Figure 3A). The expression of cell surface receptors CD80 and HLA-DR, as classical markers of the Mφ1 phenotype, and CD163, a Mφ2 phenotype marker, were evaluated by flow cytometry (Figure 3B). Results showed that LIPINOVA^®^ treatment decreased the expression of CD80 and HLA-DR in lipopolysaccharide (LPS)-stimulated Mφ1, although no significant differences were observed between treated and non-treated Mφ1 cultures. LIPINOVA^®^ had no effect on the expression of CD163 in Mφ1 cultures, and only Mφ2 cultures showed high expression levels of this marker (Figure 3C). Using the same culture conditions, we utilized qPCR to evaluate the expression of IL1β, CXCL10, and CXCL11 genes, as markers of the Mφ1 phenotype, and CD206, as a marker of the Mφ2 phenotype. Treatment of Mφ1 cultures with 50 µM LIPINOVA^®^ significantly decreased the expression of IL1β and CXCL10 genes. The expression of CXCL11 also decreased, although the differences were not significant. Notably, CD206 gene expression levels in Mφ1 macrophages were significantly elevated in cultures treated with 50 µM LIPINOVA^®^, even when compared with Mφ2 cultures (Figure 3D). Next, we used the supernatant of cell cultures to evaluate the production of proinflammatory cytokines CXCL10 and IL1β by ELISA. Mφ1 macrophages treated with 50 µM LIPINOVA^®^ produced a significantly lower amount of CXCL10. However, we did not observe changes in the production of IL1β after the addition of LIPINOVA^®^ in comparison to Mφ1 macrophages using this approach (Figure 3E). Overall, these results indicate that LIPINOVA^®^ treatment seems to suppress the expression of Mφ1 markers and to modulate the repolarization of Mφ1 towards an Mφ2-like phenotype.

### 2.4. LIPINOVA^®^ Improves Wound Healing Closure in Mice

**Figure 4:**
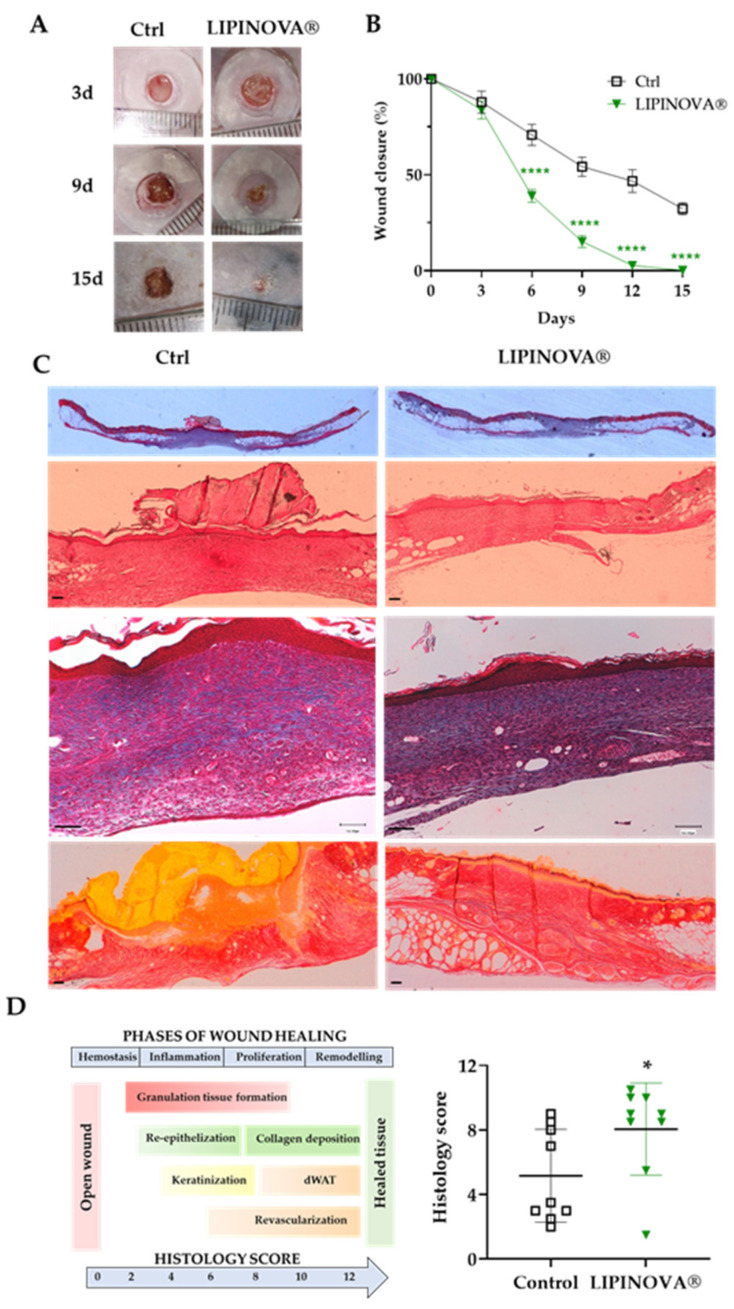
The effect of topical LIPINOVA^®^ on cutaneous wounds in *db/db* mice. (**A**) Representative images showing wounds of mice treated with saline (Ctrl) or with 50 ng LIPINOVA^®^ at different time points (day 3, 9, and 15). (**B**) Kinetics of wound closure assessed at different time points as a percentage of the initial area in saline- and LIPINOVA^®^-treated animals (*n* = 9 animals per group, 2 wounds per animal). Data are represented as mean ± SEM. Two-way ANOVA was used for statistical analysis. (**C**) Representative images of wound sections stained with H&E, Masson’s trichrome, and Picrosirius red at day 15. Images were acquired at different magnifications; scale bar = 100 µm. (**D**) Histology score represented as mean ± SD of mice treated with 50 ng LIPINOVA^®^ or saline obtained from H&E- and Masson’s trichrome-stained skin sections 15 days post-wounding. Dermal white adipose tissue (dWAT). The Mann–Whitney U test was used for statistical analysis; * *p* < 0.05, **** *p* < 0.0001.

Given the functional effects of LIPINOVA^®^ observed in vitro, we next tested its therapeutic potential in an animal model of DM2 [43]. After optimizing the cumulative dose (not shown), *db*/*db* mice were randomized into two groups: control (Ctrl; saline) and 50 ng LIPINOVA^®^. Mice were treated daily with the corresponding treatment, and the changes in wound area were measured every 3 days until sacrifice (15 days post-wounding) (Figure 4A).

Manual quantification of the wound area in all animals revealed that wounds were completely closed in the *db/db* mice treated with 50 ng LIPINOVA^®^ after 15 days, but not in the control animals (Figure 4B). Given these differences, we performed histological staining experiments to examine skin integrity using hematoxylin and eosin (H&E), Masson’s trichrome, and Picrosirius red staining. Representative images of skin sections of *db/db* mice sacrificed at day 15 after wounding are shown in Figure 4C. Wound healing was more advanced in animals treated with LIPINOVA^®^ than in the control (saline-treated) animals, as shown by thicker parakeratotic stratum corneum, less epidermal hyperplasia or cellular infiltration, and by the presence of dermal white adipose tissue in the wound area in sections stained with H&E, Masson´s trichrome, and Picrosirius red. Parameters measured included the epithelial thickness, granulation tissue, maturation of collagenous tissue, and scar elevation index (Table 2). A histology score to determine wound healing status was calculated as described in [44], which is based on different criteria using both quantitative and semi-quantitative measures, such as re-epithelization, angiogenesis, epithelial thickness, keratinization, granulation tissue formation, scar elevation index, and remodeling. We obtained a score of 5.8 ± 1.07 in control animals and 8.4 ± 0.92 in LIPINOVA^®^-treated animals (* *p* < 0.05) (Figure 4D).

### 2.5. LIPINOVA^®^ Promotes Macrophage Polarization towards an Anti-Inflammatory Profile

**Figure 5:**
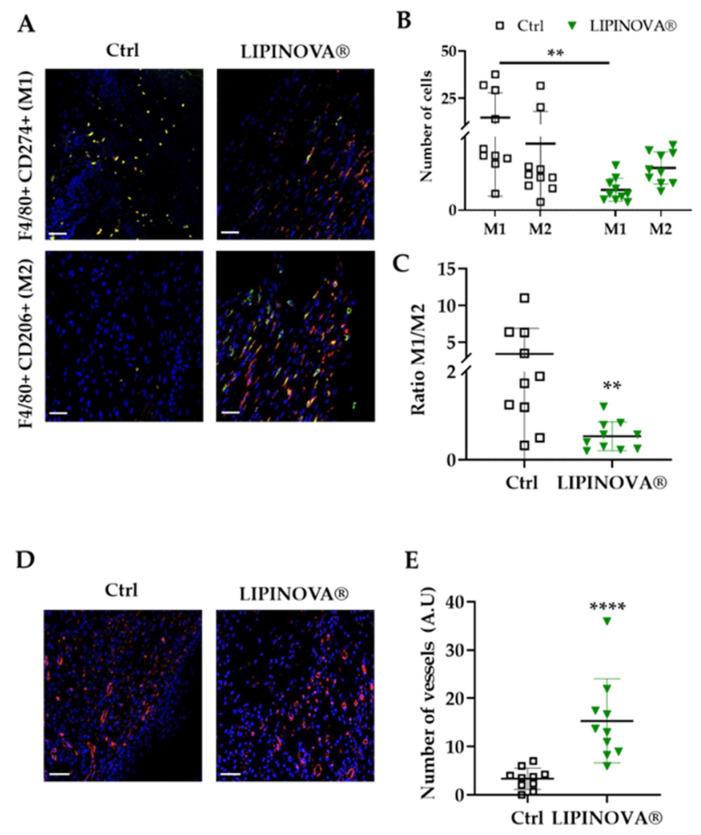
LIPINOVA^®^ promotes the modulation of Mφ1/Mφ2 phenotype and increases angiogenesis on cutaneous wounds in *db/db* mice. (**A**) Representative immunofluorescence images of wound sections stained with specific Mφ1 and Mφ2 antibodies: F4/80+ (red), CD274+ (green), and CD206+ (green). Nuclei are stained with DAPI (blue). (**B**) Quantification of the M1/M2 macrophage profile ratio. (**C**) Number of cells stained with antibodies that recognize Mφ1 and Mφ2 in control and LIPINOVA^®^ groups. (**D**) Representative images of wound sections stained with a caveolin antibody. (**E**) Quantification of the number of vessels in the wound area; scale bar = 50 µm. Data are represented as the mean ± SD, ** *p* < 0.01, **** *p* < 0.0001. *n* = 10 animals per group.

We next analyzed the profile of infiltrating macrophages in skin sections 15 days after LIPINOVA^®^ treatment. Mφ1, characterized by positive double immunostaining for F4/80+ and CD274+, and Mφ2, characterized by F4/80+ and CD206+ staining, were identified by immunohistochemistry of inflammatory infiltrates at the wound site (Figure 5A). Results showed that the number of Mφ1 at the wound site at 15 days was significantly lower (*p* < 0.05) in the group treated with LIPINOVA^®^ than in equivalent saline-treated mice. The Mφ1/Mφ2 ratio was 3.41 ± 3.46 in saline-treated mice and 0.53 ± 0.33 in LIPINOVA^®^-treated mice (Figure 5B). Moreover, the cell density of Mφ1 was higher at the wound site of saline-treated mice than in LIPINOVA^®^-treated mice, whereas the opposite was seen for Mφ2 (Figure 5C).

Because angiogenesis is detected in the last phases of the wound healing process [44], we measured vessel density at the wound sites by immunofluorescence, using an antibody to caveolin. Results showed that the number of vessels was significantly higher in the animals treated with LIPINOVA^®^ than in the saline-treated mice (Figure 5D,E), which fitted well with the results of the histology score (that included this parameter). Overall, our findings suggest that LIPINOVA^®^ induces constructive remodeling in vivo when topically administered to *db/db* mice.

## 3. Discussion

LIPINOVA^®^ is a bioactive formulation that is used as an oral supplement to favor resolution of inflammation and to date, clinical results have demonstrated its safety and efficacy without evidence of side effects [39,41]. In the present study, we explored the beneficial effects of topical administration of LIPINOVA^®^ to cutaneous ulcers in the context of diabetic pathophysiology. We found the biocompatibility and functional activity of LIPINOVA^®^ on human keratinocytes, fibroblasts, endothelial cells, and macrophages. The dietary supplement had no effect on cell survival at the doses tested, and uptake of the supplement was verified using LIPINOVA^®^-OG in dermal fibroblasts, endothelial cells, and macrophages. We observed that in vitro wound closure of keratinocytes and fibroblasts was accelerated with low doses of LIPINOVA^®^ (250 nM), but not with higher doses (50 µM). It is possible that although higher doses had no effect on cell viability, the migration response was blunted. Our findings are in accord with a previous study reporting that EPA improves the healing of fibroblast wounds in vitro [45]. Nevertheless, another study failed to detect differences in keratinocyte and fibroblast migration in cultures treated with EPA or DHA used at a concentration of 150 µM, but it is possible that the higher doses used account for these incongruities [46].

To study whether LIPINOVA^®^ could modulate macrophage populations, we added it to cultures of human peripheral blood monocytes differentiating to Mφ1 [47]. Notably, the addition of LIPINOVA^®^ reduced the number of pro-inflammatory Mφ1s and increased the number of Mφ2s, as assessed by the expression of CD206. This is particularly important, as Mφ1s release pro-inflammatory cytokines that aggravate pathological processes in chronic wounds in the absence of resolution of inflammation. We also studied the healing properties of LIPINOVA^®^ in *db/db* mice. This model is commonly used in DM2 research, as the excisional wound assay mimics the physiological processes that occur in diabetic foot ulcers [43]. We used repeated daily doses of topical LIPINOVA^®^, rather than a single dose at the beginning of the treatment, as we wanted to simulate the treatment that patients with diabetes receive, and because of the oxidative nature of PUFAs/SPMs [48]. When administered topically to open wounds in *db/db* mice, LIPINOVA^®^ induced improvements in most of the histological parameters examined, and re-epithelization was apparent in treated animals from day 6 after wounding. A role for pro-resolving lipid mediators in keratinocyte proliferation has previously been observed in vivo [49]. Moreover, interventional studies using LIPINOVA^®^ reported an increase in leukotrienes, including LTB4 and other SPMs, in the plasma of healthy subjects and patients with PAD after one month of daily supplementation [40]; thus, we assume that SPM cascades are activated in diabetic ulcers after LIPINOVA^®^ treatment. In this context, during the proliferative phase of cutaneous wound healing, the lipid mediator 10 *E*-trienoic acid (12-HHT), which is metabolized as a product of AA by the platelets, is an agonist of the leukotriene B4 receptor type 2 (BLT2) on the surface of keratinocytes. The 12-HHT/BLT2 axis promotes proliferation of the latter through a cascade of well-described biological processes, accelerating wound closure in mice [50].

Regarding the dynamics of tissue granulation, a reduction in polymorphonuclear cell infiltrates was observed 15 days post-wounding in the LIPINOVA^®^-treated animals as compared with the saline-treated controls. The concept of anti-inflammation versus pro-resolution is interesting, since the latter involves clearance of excessive accumulation of granulation tissue and macrophage efferocytosis, needed for tissue regeneration. Previous studies have shown that EPA and DHA PUFAs suppress inflammatory processes by modulating anti-inflammatory properties in macrophages [51]. In this context, the pro-resolution therapy mediated by the D-series resolvins generated from DHA has been demonstrated in the setting of diabetes, where treatment with this molecule induced efferocytosis and apoptotic clearance in diabetic wounds [25]. Additionally, in a UV radiation-challenged skin experiment, oral supplementation of ω3-PUFAs blocked the migration of Langerhans cells from the epidermis, demonstrating their role in immune processes [52]. In addition, the immune-modulation properties of resolvin D1 and DHA, at nanomolar and micromolar concentrations, respectively, were also found on adipose tissue macrophages [29]. During the process of resolution of inflammation, the main macrophage phenotype switches from pro-inflammatory M1 to pro-resolving M2. Consistent with the aforementioned studies, we observed that LIPINOVA^®^ modulated M1/M2 polarization. Although treatment with this dietary supplement increased the number of M2 macrophages, the main effect was the reduction in the M1 subtype, both in vitro and in vivo, pointing to an important effect on macrophage differentiation.

LIPINOVA^®^ treatment in *db/db* mice promoted the formation of mature dense connective tissue characterized by the presence of collagen bundles, while preventing excessive scarring, as assessed by the histology score in the Picrosirius-stained sections. These results are in line with other reported findings [11], which revealed that oral administration of ω-3 fatty acids promoted epithelial healing, while reducing collagen deposition in an ear punch full-thickness wound healing model in mice on a high-fat diet. Similarly, DHA supplementation reduced α-smooth muscle actin-positive matrix-producing cells in a model of experimental hepatic fibrosis [53], and had an anti-fibrogenic effect in a murine model of liver injury [18]. Interestingly, the latter study showed that cirrhotic livers are depleted of DHA, and this depletion correlated with the progression of the disease.

We found that wounds treated with LIPINOVA^®^ showed improved angiogenesis. This observation is in line with previous reports demonstrating the angiogenic potential of DHA and EPA in mesenchymal stem cells for wound healing applications [54], as well as the increase in the number of blood vessels in the cutaneous tissue of wounds after fish oil treatments [12,21]. However, precise comparisons among the studies are difficult, as the composition of fish oils, including the amount of PUFAs (EPA/DHA) and SPMs, is different.

In summary, we demonstrate the potential of LIPINOVA^®^ to promote healing processes by reducing immune inflammatory reactions at the wound site and inducing angiogenesis and M1/M2 polarization in wounded diabetic mice. Topical administration of this marine oil was sufficient to accelerate wound closure, indicating the importance of SPMs in this process. Of note, while other studies tested PUFAs by oral or systemic administration to treat cutaneous ulcers, we used topical administration instead. However, since the dietary supplement was administered to open wounds, we cannot discard the entrance of different components of LIPINOVA^®^ into open circulation, and this should be tested in future studies. As LIPINOVA^®^ has been used to reduce chronic pain when administered orally, our preclinical findings could serve as a basis for developing clinical trials in diabetic patients with foot ulcers.

## 4. Materials and Methods

### 4.1. Ethical Statements

Human primary cultures and blood were obtained from healthy donors who gave their informed consent. The study was conducted in accordance with the Declaration of Helsinki, and the protocol was approved by the Ethics Committee of The Hospital La Fe Universitari i Politècnic, Valencia, Spain (project and protocol identification 2015/0097-CEIM: 2016040405).

All animal procedures were approved by institutional ethical and animal care committees (reference number 2019/VSC/PEA/0257 for the wound healing experiment).

### 4.2. LIPINOVA^®^ Manufacturing Process and SPM Analysis

The LIPINOVA^®^-11TG batch (Solutex GC, Alcobendas, Spain) was used in the present study. The dietary supplement was generated from semi-refined fish oil (from anchovies and sardines). The first step in the process was esterification, followed by a concentration step (distillation). The intermediates generated in the process were processed using CO_2_ in a supercritical fluid extraction process, which benefits from the fact that CO_2_ in supercritical conditions can operate at moderate temperatures, without undue product stress, preventing further degradation of the products due to the inert properties of the oxidation. The process resulted in a fraction with standardized levels of 17-HDHA (80–400 mg/kg), 18-HEPE (50–400 mg/kg), 14-HDHA (40–200 mg/kg), EPA (100–300 mg/g), and DHA (200–450 mg/g). An enzymatic esterification step was then performed to substitute the ethyl ester for a glyceryl ester, by combining the three fatty acids with the three binding positions of the glycerol molecule (tri-alcohol), to re-establish the triglyceride structure. A deodorization step was then performed to reduce the volatile components of the product and, consequently, its odor, improving the comfort of its use as an oral supplement. The last step was a homogenization step, with an added quantity of natural antioxidants (such as a mixture of tocopherols), which improves the oxidative stability of the final product.

The SPM analysis of LIPINOVA was carried out, as previously described in [55], with some modifications. Briefly, samples were analyzed by liquid chromatography (Agilent 1260, San Jose, CA, USA) coupled with electrospray ionization on a triple quadrupole mass spectrometer (Agilent 6410, San Jose, CA, USA). For analysis, 10 μL of the extract was injected. The auto sampler was cooled at 10 °C. Chromatographic separation was achieved on an zorbax eclipse plus (2.1 × 50 mm, 1.8 µm particles; Agilent) column using a flow rate of 0.5 mL/min at room temperature during an 11 min gradient (0–4 min, 58% B; 4–10 min, 100% B; 10–10.5 min, 58% B; 10.5–11 min, 58% B), while using the solvents A, 0.01% acetic acid in water, and B, 0.01% acetic acid in methanol. Electrospray ionization was performed in the negative ion mode using N2 at a pressure of 35 psi for the nebulizer with a flow of 10 L/min and a temperature of 300 °C, respectively. The sheath gas temperature was 350 °C with a flow rate of 8 L/min. The capillary was set at 4000 V.

### 4.3. Cell Culture of Adherent Primary Cultures

Primary cultures of human dermal fibroblasts and keratinocytes were obtained from skin samples of patients undergoing abdominal surgery, as described in [56]; cells were cultured in low-glucose Dulbecco’s Modified Eagle’s Medium (DMEM) and high-glucose DMEM (both from Sigma-Aldrich, St Louis, MO, USA), respectively, supplemented with 10% fetal bovine serum (FBS, ThermoFisher Scientific, Waltham, MA, USA), along with 100 U mL^−1^ penicillin and 100 µg mL^−1^ streptomycin (P/S, Millipore, Bedford, MA, USA). Primary cultures of human umbilical cord vein endothelial cells (HUVEC, ATCC CRL-1730) were obtained from Lonza (Basel, Switzerland) and were grown in Endothelial Cell Growth Medium-2 (EGM-2) BulletKit^TM^ (Lonza). All cells were maintained in a humidified atmosphere at 37 °C and 5% CO_2_.

### 4.4. Isolation and Culture of Peripheral Blood Monocytes

Monocytes (CD14+ cells) were isolated from buffy coats of healthy donors after informed consent. Blood was diluted in HBSS 1× (Gibco, ThermoFisher Scientific) and centrifuged. The middle layer was diluted in HBSS 1×, and peripheral blood mononuclear cells (PBMCs) were isolated using Histopaque^®^-1077 (Sigma-Aldrich) density gradient centrifugation. Isolated PBMCs were resuspended in RPMI (ThermoFisher Scientific) supplemented with 10% FBS, 1 mM pyruvate, 2 mM glutamine and 10 μg mL^−1^ ciprofloxacin (all from Sigma-Aldrich), and were allowed to adhere on culture plates for 1–2 h. Non-adherent cells were washed, and adherent monocytes were then cultured in RPMI. Monocytes were differentiated to Mφ1 or Mφ2 macrophages by adding 5 ng mL^−1^ of recombinant human granulocyte macrophage-colony stimulating factor (rhGM-CSF, Invitrogen, Waltham, MA, USA) or 20 ng mL^−1^ recombinant human macrophage-colony stimulating factor (rhM-CSF, Invitrogen), respectively. Cytokine stimulation was repeated on day 3. On day 5, 10 ng mL^−1^ of LPS (Invitrogen) and 20 ng mL^−1^ of IFNɣ (R&D Systems, Minneapolis, MN, USA) were added to the Mφ1 macrophages, whereas 10 ng mL^−1^ of LPS and 40 ng mL^−1^ of IL4 (PeproTech, London, UK) were added to the Mφ2 macrophages. Under Mφ1 conditions, 250 nM or 50 μM of LIPINOVA^®^ were added on days 0, 3, and 5 of the differentiation protocol.

### 4.5. Preparation of LIPINOVA^®^ for In Vitro Assays

Due to the instability of LIPINOVA^®^ in an oxygen atmosphere and its low solubility, it was prepared in DMEM supplemented with 10% fatty acid-free bovine serum albumin (FAF-BSA, Sigma-Aldrich) to a 10 mM stock concentration. FAF-BSA was mixed with DMEM and stirred for 3 h at room temperature and then filtered through a 0.22-µm filter. Finally, 1 mM LIPINOVA^®^ (8.53 mg/mL) was prepared and stirred again for 16 h at 37 °C. LIPINOVA^®^ stocks were freshly prepared for each experiment. For internalization studies, LIPINOVA^®^ was labeled with Oregon green (LIPINOVA^®^-OG) by Dr. María Jesús Vicent (Polymer therapeutics laboratory, Centro de Investigación Príncipe Felipe, Valencia, Spain).

### 4.6. Cytotoxicity Assays

Fibroblasts and keratinocytes were seeded in 96-well plates at 1200 and 1600 cells cm^−2^, respectively. After 24 h, cells were incubated with the following concentrations of LIPINOVA^®^ (µM): 1000, 500, 250, 125, 62, 31, and 0. Cytotoxicity was evaluated using the water-soluble tetrazolium-8-[2-(2-methoxy-4-nitrophenyl)-3-(4-nitrophenyl)-5-(2,4-disulfophenyl)-2H-tetrazolium] monosodium salt (CCK-8) assay (Sigma-Aldrich). Absorbance was measured at 450 nm on a Perkin-Elmer Victor3 1420 Multilabel Counter microplate reader (PerkinElmer Inc., Waltham, MA, USA). Three independent experiments were performed in triplicate.

### 4.7. Flow Cytometry

Differentiated macrophages were washed and incubated with a blocking solution (PBS containing 1% normal mouse serum) for 10 min. Thereafter, cells were incubated with saturating amounts of fluorochrome-conjugated antibodies at 4 °C for 1 h and then washed. The human antibodies used were as follows: anti-CD80 (APC, BD Biosciences, Erebodegem, Belgium), anti-CD163 (PerCP-Cy, BD Biosciences, GHI/61), and anti-HLA-DR (FITC, Miltenyi Biotech, AC122, Bergisch Gladbach, Germany). Results were analyzed on a BD FACSCanto II flow cytometer using FlowJo^®^ software (FlowJo LLC, BD, Franklin Lakes, NJ, USA).

### 4.8. Real Time Quantitative PCR

RNA was obtained with the RNeasy Plus Mini Kit (Qiagen, Dusseldorf, Germany) and cDNA was obtained by reverse transcription using the PrimeScript RT Reagent Kit (Takara, Kusatsu, Japan). RT-qPCR was performed with specific sense (F) and anti-sense (R) primers and the RT-SYBR™ Green PCR Master Mix (Applied Biosystems, Waltham, MA, USA). Multi-well plates of 384 wells were run on a Viia 7 PCR System (Applied Biosystems). Primers used were as follows: *hGAPDH* CCCCTCTGCTGATGCCCCA (F) and TGACCTTGGCCAGGGGTGCT (R); *hCXCL10* TGCAAGCCAATTTTGTCCACGTGT (F) and GCAGCCTCTGTGTGGTCCATCC (R); *hCXCL11* TGTCTTTGCATAGGCCCTGGGGT (F) and AGCCTTGCTTGCTTCGATTTGGGA (R); hIL1β AGGCACAAGGCACAACAGGCT (F) and AACAACTGACGCGGCCTGCC (R); and hCD206 ACCTGCGACAGTAAACGAGG (F) and TGTCTCCGCTTCATGCCATT (R).

### 4.9. Measurement of Cytokines by Enzyme-Linked Immunosorbent Assay (ELISA)

Macrophages were treated and differentiated in vitro. Supernatants were harvested and used to measure the levels of CXCL10 and IL1-β. The quantification of these pro-inflammatory cytokines was conducted by commercial ELISA kits (Invitrogen, Waltham, MA, USA), according to the manufacturer’s instructions.

### 4.10. In Vitro Wound Healing Assay

Fibroblasts and keratinocytes were seeded in 12-well culture plates, as previously described in [57]. When confluence was reached, cells were scraped, washed, and treated with 250 nM or 50 µM of LIPINOVA^®^. Images of the scraped zone were captured every 6 h for 48 h using a Leica DMi8 Platform live cell microscope with controlled CO_2_ and temperature (Leica Microsystems, Wetzlar, Germany). Cell migration was evaluated using ImageJ software (NIH, Bethesda, MD, USA). Three independent experiments were performed in triplicate.

### 4.11. Animals

Adult type 2 diabetes (*db/db*) mice (R/BKS.CG-M+/+LEPR DB/J, age 8 weeks) were purchased from Charles River Laboratories Inc. (Wilmington, MA, USA), and were housed in a barrier facility with a controlled ambient temperature and under a 14:10 light-dark cycle.

### 4.12. In Vivo Wound Healing

The day before the dorsal wound generation, mice were anesthetized by isoflurane inhalation and the dorsal area was shaved with a depilatory cream. Two wounds were made on the dorsal skin of each animal using a 6 mm punch biopsy tool, and a silicone splint was attached to prevent skin contraction [25]. Mice were topically treated daily with saline (control) or 50 ng of LIPINOVA^®^ (*n* = 10 animals in each group). Wound areas were measured daily. Mice were sacrificed on day 15 and wound tissues were excised and processed for histology.

### 4.13. Histology and Morphometric Analysis

Skin biopsies were fixed in 70% ethanol, embedded in paraffin, and sectioned in transverse sections of 5 µm, as described in [56]. Sections were stained with H&E, Masson’s trichrome, and Picrosirius red (all from Sigma-Aldrich) or were incubated with specific antibodies for immunohistochemistry. Sections were observed and images were acquired using a light microscope for stained sections (Leica DMD 108) and a confocal microscope for immunohistochemistry experiments (Leica TCS-SP5-AOBS).

Inflammatory infiltrates of H&E-stained sections from the different treated groups and a histology score of wound healing was assessed by researchers blinded to the group categorization of the animals, as described in [44]. The scar elevation index was calculated measuring the dermal thickness at the site of the wound (DTW) or in healthy non-wounded (DTH) areas, as follows: (DTH-DTW)/DTW. Collagen deposition was measured in Picrosirius red-stained sections using ImageJ software. The red area of each section was measured and normalized against the total area of the image. For each animal, wound measures were normalized against healthy skin. Based on these results, a score between 0 and 2 was given to each animal.

### 4.14. Analysis of Macrophage Mφ1/Mφ2

To analyze Mφ1 and Mφ2 macrophages, skin sections were stained for F4/80, CD274, and CD206 to quantify the macrophage phenotype surrounding the implant site. Immunolabeling was performed with antibodies against F4/80 (1:50 dilution; Abcam, Cambridge, UK), combined either with CD274 (Mφ1) (1:100 dilution; Novus Biologicals, Littleton, CO, USA) or CD206 (Mφ2) (1:200 dilution; Abcam), followed by detection with Alexa-488-conjugated secondary antibodies (1:200 dilution; Invitrogen). Quantification was performed in mice sacrificed 15 days post-treatment. A total of four serial skin sections were prepared, and four images of the zone of the implant were taken per section with a 20× objective. Immunofluorescent images were acquired with a Leica TCS-SP5-AOBS microscope (Leica Microsystems, Wetzlar, Germany), the immunopositive cells were quantified, and the ratio of Mφ1/Mφ2 macrophages was calculated in each field.

### 4.15. Analysis of Blood Vessel Density

Angiogenesis was evaluated by staining with an antibody against caveolin (1:200 dilution; Cell Signaling, Danvers, MA, USA), followed by detection with an Alexa 488 (1:200 dilution; Invitrogen). Images were acquired with a Leica DM2500 fluorescence microscope (Leica Microsystems); four images of the zone of the implant were taken per section with a 20× objective and then analyzed with ImageJ software.

### 4.16. Statistical Analysis

Data are represented as mean ± SD. Groups were compared by one-way and two-way ANOVA and post hoc analysis, when necessary. Analyses were conducted with GraphPad Prism 5^®^ software (San Diego, CA, USA). Differences were considered statistically significant at *p*-value < 0.05, with a 95% confidence interval.

## Figures and Tables

**Table 1:** Essential fatty acids in the Lipinova-11TG batch. Data are represented as % area.

Fatty Acids		% Area
Myristic	C14:0	0.44
Myristoleic	C14:1	n.d.
Pentadecanoic	C15:0	n.d.
Palmitic	C16:0	1.31
Palmitoleic	C16:1 n7	0.61
Hexadecaenoic	C16:4 n1	0.23
Margaric	C17:0	0.06
Margaroleic	C17:1	0.12
Stearic	C18:0	1.00
Oleic	C18:1 n9	2.05
Vaccenic	C18:1 n7	0.65
Linoleic	C18:2 n6	0.25
Gamma-linolenic	C18:3 n6	0.08
Linolenic	C18:3 n3	0.15
Stearidonic	C18:4 n3	0.67
Arachidic	C20:0	0.73
Eicosenoic	C20:1 n9	2.32
Gondonic	C20:1 n7	0.38
Arachidonic	C20:4 n6	0.96
Eicosatetraenoic	C20:4 n3	1.13
Eicosapentaenoic	C20:5 n3	20.08
Behenic	C22:0	0.90
Erucic	C22:1 n11	2.84
Adrenic	C22:4 n6	0.52
Docosapentaenoic	C22:5 n6	0.98
Docosapentaenoic	C22:5 n3	7.57
Lignoceric	C24:0	0.46
Docosahexaenoic	C22:6 n3	45.47
Nervonic	C24:1 n9	0.23
Total ω3		75.07
Total ω6		0.98
Total ω9		4.60
SFA		4.90
MUFA		9.20
PUFA		78.09

SFA: saturated fatty acids; MUFA: monounsaturated fatty acids; PUFA: polyunsaturated fatty acids.

**Table 2:** Histological parameters analyzed in mice treated with saline or with 50 ng LIPINOVA^®^. The study was performed at 15 days post-wounding.

Factors Evaluated (at 15 Days)	Ctrl	LIPINOVA^®^
Epithelial thickness (µm)	72.86 ± 27.80	62.19 ± 19.48
Granulation tissue (µm)	439.44 ± 133.95	310.14 ± 125.58
Scar elevation index	1.35 ± 0.12	1.29 ± 0.20
Re-epithelization (0–2)	0.89 ± 1.05	1.78 ± 0.71
Keratinization (0–2)	1.11 ± 0.93	1.78 ± 0.71
Collagen deposition (0–2)	0.70 ± 0.28	0.66 ± 0.19
Remodeling (0–2)	0.78 ± 0.44	0.89 ± 0.25

Ctrl: control group. Data are represented as mean ± SD.

## Data Availability

Not applicable.
